# Preterm birth, birth weight, and subsequent risk of female breast cancer

**DOI:** 10.1038/sj.bjc.6601357

**Published:** 2003-10-28

**Authors:** M Kaijser, O Akre, S Cnattingius, A Ekbom

**Affiliations:** 1 Clinical Epidemiology Unit, Department of Medicine at Karolinska Hospital, M9:01, Karolinska Sjukhuset, S-171 76 Stockholm, Sweden; 2 Department of Medical Epidemiology, Karolinska institutet, Box 281, S-171 77 Stockholm, Sweden

**Keywords:** preterm birth, birth weight, breast cancer, cohort study

## Abstract

We have previously found an increased risk of breast cancer among women born preterm. To confirm or refute the results, an enlarged study was conducted. The results from this study do not confirm the initial findings and suggest that preterm birth can be ruled out as a risk factor for breast cancer.

Oestrogens are fundamental hormones in breast cancer aetiology, and it has been suggested that the surge in oestrogens during pregnancy may influence subsequent breast cancer risk in the offspring (Trichopoulos, 1990).

Infants born extremely preterm, that is before 28 weeks of gestation, has been found to have high levels of oestrogens postnatally (Sedin *et al*, 1985). In a study of women born between 1925 and1932, we found that birth before 32 weeks of gestation constituted a strong risk factor for adult breast cancer (Ekbom *et al*, 2000). The study, however, was hampered by low statistical power. We therefore conducted an enlarged study of the association between preterm birth and female breast cancer.

## MATERIALS AND METHODS

We identified the study subjects by examining manually all birth records for the period 1925–1949 from Uppsala University Hospital, Sundsvalls County Hospital, and two major delivery units in Stockholm (Allmänna BB and Södra BB). Approximately 250 000 birth records were examined. Female subjects born before 35 completed weeks of gestation according to date of last menstrual period, and females born after 35 weeks with a birth weight of less than 2000 g were included. At the point of initial inclusion into the cohort, data on pregnancy and delivery, such as date of last menstrual period and birth weight, was recorded.

By use of the National Registration Number, the cohort was followed up in the Swedish Register of Population and Population Changes from 1 January 1958 to 31 December 1998 or to death or emigration prior to this time. Subjects who deceased before 1958 were excluded, since incident cancers could not be identified with any completeness prior to the initiation of the Swedish Cancer Register. We identified cases of cancer by linking our subjects to the Swedish Cancer Register. The completeness of this register is over 98% (Mattsson and Wallgren, 1984).

We used last menstrual period to estimate gestational duration. We categorised gestational duration into three groups: 32 completed weeks or less, 33–34 weeks, and 35 weeks or more. Birth weight was categorised into less than 2000 g, 2000–2999 g, and 3000 g or more. Since an unreasonably high birth weight for a short duration of gestation indicated an underestimation of gestational duration or an overestimation of birth weight, we excluded subjects born before 32 weeks of gestation with a birth weight of 3000 g or more (*n*=155), and subjects with a gestational duration of 33–34 weeks with a birth weight of 3600 g or more (*n*=94).

We calculated standardised incidence ratios (SIR) for all female cancer, female breast cancer, and early onset female breast cancer using age, sex, and time-specific cancer incidence rates for the Swedish population during the period 1958–1998 provided by the Cancer Registry. We analysed all cancer occurrences as well as the first cancer occurrence. As these estimates were essentially the same, we only present the results from the analysis of first cancer occurrence.

## RESULTS

There were 1483 women who met the eligibility criteria and of these, 1208 were born before 35 weeks of gestation and 275 were born after week 35 with a birth weight of less than 2000 g (Table 1

**Table 1:** Study subjects by category, gestational duration, and birth weight

**Study subjects**	**No.**	**Percentage** (**%)**
All	1483	100
		
*Small-for-gestational-age*^a^	275	18.5
		
*Preterm*		
All	1208	81.5
⩽32 weeks	514	34.7
33–34 weeks	694	46.8
		
*Birth weight*		
0.5–1.99 kg	703	47.4
2–2.99 kg	691	46.6
⩾3 kg	89	6.0
		
*Gestational duration by birth weight*		
⩽32 weeks		
0.5–1.99 kg	292	19.7
2–2.99 kg	222	15.0
33–34 weeks		
0.5–1.99 kg	136	9.2
2–2.99 kg	469	31.6
⩾3 kg	89	6.0

a Birth weight<2000 g and gestational duration>35 weeks.

).

The overall risk of cancer among the women was not increased (Table 2

**Table 2:** Standardised incidence ratios (SIR) and 95% confidence intervals (95% CI) for all female cancer, female breast cancer and female early onset breast cancer^a^ by category, gestational duration, and birth weight

	**All cancers**				**All breast cancer**				**Early oneset breast cancer**^**a**^			
	**Obs**	**Exp**	**SIR**	**95% CI**	**Obs**	**Exp**	**SIR**	**95% CI**	**Obs**	**Exp**	**SIR**	**95% CI**
*Small-for-gestational-age*^b^	20	26.7	0.75	0.46–1.16	10	9.2	1.09	0.52–2.01	4	3.8	1.06	0.29–2.71
												
*Preterm*												
All	127	112.2	1.13	0.94–1.35	39	39.4	0.99	0.70–1.35	19	16.8	1.13	0.68–1.77
⩽32 weeks	55	47.8	1.15	0.87–1.50	18	16.7	1.08	0.64–1.70	10	7.1	1.40	0.67–2.57
33–34 weeks	72	64.4	1.12	0.88–1.41	21	22.7	0.92	0.57–1.41	9	9.7	0.93	0.43–1.77
												
*Birth weight*^c^												
0.5–1.99 kg	45	39.9	1.13	0.82–1.51	16	14.0	1.14	0.65–1.85	9	5.9	1.53	0.70–2.91
2–2.99 kg	63	64.7	0.97	0.75–1.25	16	22.7	0.71	0.40–1.15	7	9.7	0.72	0.29–1.49
⩾3 kg	19	7.6	2.51	1.51–3.91	7	2.7	2.55	1.03–5.25	3	1.2	2.46	0.51–7.19
												
*Gestational duration by birth weight*												
⩽32 weeks												
0.5–1.99 kg	32	26.9	1.19	0.81–1.68	13	9.4	1.38	0.73–2.36	8	4.0	1.99	0.86–3.92
2–2.99 kg	23	20.9	1.10	0.70–1.65	5	7.3	0.69	0.22–1.60	2	3.1	0.64	0.08–2.31
33–34 weeks												
0.5–1.99 kg	13	13.1	1.00	0.53–1.70	3	4.6	0.65	0.13–1.91	1	1.8	0.54	0.01–3.01
2–2.99 kg	40	43.7	0.91	0.65–1.25	11	15.4	0.72	0.36–1.28	5	6.6	0.76	0.25–1.77
⩾3 kg	19	7.6	2.51	1.51–3.91	7	2.7	2.55	1.03–5.25	3	1.2	2.46	0.51–7.19

a Onset before 50 years of age.

b Birth weight<2000 g and gestational duration>35 weeks.

c Among women born before 35 weeks of gestation.

). The risk of breast cancer was neither associated with preterm birth nor with low birth weight, but a birth weight of more than 3000 g was associated with an increased risk of breast cancer (Table 2). This increase in breast cancer risk was not dependent on age at onset.

## DISCUSSION

This study is, to our knowledge, the largest cohort study hitherto conducted on long-term cancer risk among women born preterm or with low birth weight. Neither preterm birth nor low birth weight were associated with an increased risk for cancer overall or for breast cancer.

We have previously reported an increased risk for breast cancer among women born before 32 weeks of gestation(Ekbom *et al*, 1997; Ekbom *et al*, 2000). Other studies of the association between gestational duration and breast cancer have been mostly negative (Le Marchand *et al*, 1988; Michels *et al*, 1996; Sanderson *et al*, 1996; Sanderson *et al*, 1998), but these studies have categorised preterm birth either as a dichotomous variable(Le Marchand *et al*, 1988; Sanderson *et al*, 1996; Sanderson *et al*, 1998), or as preterm birth of more or less than 4 weeks(Michels *et al*, 1996). Since the majority of preterm infants are born after 32 weeks of gestation, the number of infants born before 32 weeks has been low. In a recent case–control study, however, a reduced risk for breast cancer was found among women born before 32 weeks (Innes *et al*, 2000), and as suggested by the present data, our previous results were either due to chance, or confined to a subset of women with specific characteristics.

Consistent with other studies (Potischman and Troisi, 1999; Innes *et al*, 2000; Stavola *et al*, 2000), we found a positive association between birth weight and breast cancer risk, and this association was independent of age at diagnosis. Some studies have reported the association between birth weight and breast cancer to be J-shaped, with an increased risk also among women with a very low birth weight (Potischman and Troisi, 1999; Innes *et al*, 2000). According to the present data, low birth weight is not a major risk factor for breast cancer.

When Trichopoulos (1990) put forward his hypothesis of early environmental exposures, the proposed mechanism was oestrogen exposure. Since then, a vast number of studies have supported his hypothesis, although hormones other than oestrogen may be instrumental (Potischman and Troisi, 1999). Our primary study of preterm women was originally initiated because of reports of an ovarian hyper stimulation syndrome among female infants born before 28 weeks of gestation, which lead to high levels of oestrogens perinatally (Tapanainen *et al*, 1981; Sedin *et al*, 1985). The increase in breast cancer risk was interpreted as indicating an important role of early oestrogen exposure (Ekbom *et al*, 2000). We conclude from the present study that there is no substantial increase in breast cancer risk among women born preterm. We cannot, however, draw conclusions about the role of hormones or ovarian hyper stimulation syndrome on basis of our data.

The use of self-reported date of last menstrual period leads to some misclassification in the calculation of gestational duration. The nature of this misclassification is both nondifferential and systematic, the latter with a tendency to overestimate gestational duration(Henriksen *et al*, 1995; Savitz *et al*, 2002). By exclusion of subjects whose birth weights indicated an underestimation of gestational duration, however, misclassification was reduced, and a systematic overestimation of gestational duration can only lead to a more conservative inclusion of subjects in the cohort. The main limitation of the study is that study subjects were born between 1925 and 1949, but the follow-up did not start until 1958. The cohort will therefore consist of women born more than 50 years ago who survived beyond childhood. Given the increasing proportion of survivors among infants born preterm or small-for-gestational-age, other disease patterns may occur among infants born preterm or small-for-gestational-age today.

In conclusion, we found that neither preterm birth, nor low birth weight, are associated with an increased risk for breast cancer.

## References

[bib1] Ekbom A, Erlandsson G, Hsieh C, Trichopoulos D, Adami HO, Cnattingius S (2000) Risk of breast cancer in prematurely born women. J Natl Cancer Inst 92: 840–841 10814680 10.1093/jnci/92.10.840

[bib2] Ekbom A, Hsieh CC, Lipworth L, Adami HQ, Trichopoulos D (1997) Intrauterine environment and breast cancer risk in women: a population-based study. J Natl Cancer Inst 89: 71–76 8978409 10.1093/jnci/89.1.71

[bib3] Henriksen TB, Wilcox AJ, Hedegaard M, Secher NJ (1995) Bias in studies of preterm and postterm delivery due to ultrasound assessment of gestational age. Epidemiology 6: 533–537 8562631 10.1097/00001648-199509000-00012

[bib4] Innes K, Byers T, Schymura M (2000) Birth characteristics and subsequent risk for breast cancer in very young women. Am J Epidemiol 152: 1121–1128 11130617 10.1093/aje/152.12.1121

[bib5] Le Marchand L, Kolonel LN, Myers BC, Mi MP (1988) Birth characteristics of premenopausal women with breast cancer. Br J Cancer 57: 437–439 3390382 10.1038/bjc.1988.99 PMC2246557

[bib6] Mattsson B, Wallgren A (1984) Completeness of the Swedish Cancer Register. Non-notified cancer cases recored on death certificates in 1978. Acta Radiol Oncol: 305–313 6095600 10.3109/02841868409136026

[bib7] Michels KB, Trichopoulos D, Robins JM, Rosner BA, Manson JE, Hunter DJ, Colditz GA, Hankinson SE, Speizer FE, Willett WC (1996) Birthweight as a risk factor for breast cancer (see comments). Lancet 348: 1542–1546 8950880 10.1016/S0140-6736(96)03102-9

[bib8] Potischman N, Troisi R (1999) *In-utero* and early life exposures in relation to risk of breast cancer. Cancer Causes Control 10: 561–573 10616825 10.1023/a:1008955110868

[bib9] Sanderson M, Williams MA, Daling JR, Holt VL, Malone KE, Self SG, Moore DE (1998) Maternal factors and breast cancer risk among young women. Paediatr Perinat Epidemiol 12: 397–407 9805713 10.1046/j.1365-3016.1998.00133.x

[bib10] Sanderson M, Williams MA, Malone KE, Stanford JL, Emanuel I, White E, Daling JR (1996) Perinatal factors and risk of breast cancer. Epidemiology 7: 34–37 8664398 10.1097/00001648-199601000-00007

[bib11] Savitz DA, Terry Jr JW, Dole N, Thorp Jr JM, Siega-Riz AM, Herring AH (2002) Comparison of pregnancy dating by last menstrual period, ultrasound scanning, and their combination. Am J Obstet Gynecol 187: 1660–1666 12501080 10.1067/mob.2002.127601

[bib12] Sedin G, Bergquist C, Lindgren PG (1985) Ovarian hyperstimulation syndrome in preterm infants. Pediatr Res 19: 548–552 3892471 10.1203/00006450-198506000-00009

[bib13] Stavola BL, Hardy R, Kuh D, Silva IS, Wadsworth M, Swerdlow AJ (2000) Birthweight, childhood growth and risk of breast cancer in a British cohort. Br J Cancer 83: 964–968 10970703 10.1054/bjoc.2000.1370 PMC2374673

[bib14] Tapanainen J, Koivisto M, Vihko R, Huhtaniemi I (1981) Enhanced activity of the pituitary-gonadal axis in premature human infants. J Clin Endocrinol Metab 52: 235–238 6780586 10.1210/jcem-52-2-235

[bib15] Trichopoulos D (1990) Hypothesis: does breast cancer originate *in utero*? (see comments). Lancet 335: 939–940 1970028 10.1016/0140-6736(90)91000-z

